# 4,5,6,7-Tetra­chloro-*N*-(2-fluoro­phen­yl)phthalimide

**DOI:** 10.1107/S1600536810032575

**Published:** 2010-08-28

**Authors:** Xian-Shu Fu, Xiao-Ping Yu, Wei-Min Wang, Fang Lin

**Affiliations:** a College of Life Sciences, China Jiliang University, Hangzhou 310018, People’s Republic of China

## Abstract

In the title compound, C_14_H_4_Cl_4_FNO_2_, the benzene ring and the phthalimide plane are nearly planar, the maximum deviations being 0.005 (2) and 0.010 (2) Å, respectively, but the mol­ecule as a whole is not planar: the dihedral angle between the two planar ring systems is 68.06 (10)°. A short Cl⋯O contact of 2.914 (2) Å exists in the crystal structure.

## Related literature

The title compound is an inter­mediate in the synthesis of organic electroluminescent materials; see: Han & Kay (2005 ▶). For details of the synthesis, see: Valkonen *et al.* (2007 ▶); Barchin *et al.* (2002 ▶). For related structures, see: Xu *et al.* (2006 ▶); Fu *et al.* (2010*a* ▶,*b* ▶,*c* ▶).
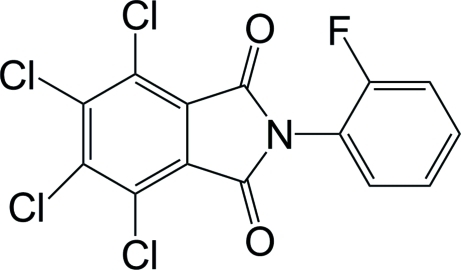


## Experimental

### 

#### Crystal data

C_14_H_4_Cl_4_FNO_2_
*M* *_r_* = 378.98
Monoclinic, 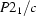

*a* = 12.032 (2) Å
*b* = 13.393 (3) Å
*c* = 8.7244 (17) Å
β = 95.33 (3)°
*V* = 1399.8 (5) Å^3^
*Z* = 4
Mo *K*α radiation
μ = 0.86 mm^−1^
*T* = 113 K
0.22 × 0.20 × 0.16 mm

#### Data collection

Rigaku Saturn CCD area-detector diffractometer
Absorption correction: multi-scan (*CrystalClear*; Rigaku, 2005 ▶) *T*_min_ = 0.833, *T*_max_ = 0.875
9870 measured reflections
2462 independent reflections
2120 reflections with *I* > 2σ(*I*)
*R*_int_ = 0.027

#### Refinement

*R*[*F* ^2^ > 2σ(*F* ^2^)] = 0.032
*wR*(*F* ^2^) = 0.085
*S* = 1.14
2462 reflections
199 parameters
H-atom parameters constrained
Δρ_max_ = 0.55 e Å^−3^
Δρ_min_ = −0.54 e Å^−3^

### 

Data collection: *CrystalClear* (Rigaku, 2005 ▶); cell refinement: *CrystalClear*; data reduction: *CrystalClear*; program(s) used to solve structure: *SHELXS97* (Sheldrick, 2008 ▶); program(s) used to refine structure: *SHELXL97* (Sheldrick, 2008 ▶); molecular graphics: *SHELXTL* (Sheldrick, 2008 ▶); software used to prepare material for publication: *SHELXTL*.

## Supplementary Material

Crystal structure: contains datablocks I, global. DOI: 10.1107/S1600536810032575/bv2150sup1.cif

Structure factors: contains datablocks I. DOI: 10.1107/S1600536810032575/bv2150Isup2.hkl

Additional supplementary materials:  crystallographic information; 3D view; checkCIF report

## Figures and Tables

**Fig. 1.**
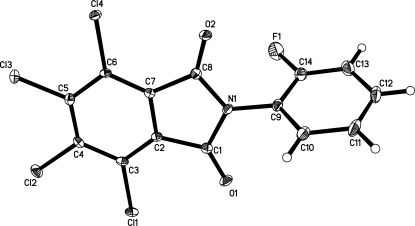
View of the molecule of showing the atom-labelling scheme. Displacement ellipsoids are drawn at the 30% probability level.

## supplementary crystallographic information

### Comment

The title compound is a key intermediate in the synthesis of organic electro-luminescent materials. The emission of light by organic molecules exposed to an electric field has been wide investigated in both an academic and industrial context. (Han & Kay, 2005).

The molecular structure of the title compound is illustrated in Fig. 1. In the title compound, the two rings are nearly planar, the maximum deviations being 0.005 (2) and 0.010 (2) Å, respectively, but the molecule as a whole is not planar. The dihedral angle between the benzene ring and the phthalimide plane is 68.06 (10) °, which is greater than 59.95 (4) ° found in a related compound *N*-(2-fluorophenyl)phthalimide (Xu  *et al.,* 2006). A short Cl···O contact of 2.914 (2) Å exists in the crystal structure.

### Experimental

An acetic acid solution of 4,5,6,7-tetrachlorophthalic anhydride (28.6 g, 100 mmol) and 2-fluoroaniline (9.65 ml, 100 mmol) was refluxed overnight, and then filtered. The crude produce was recrystallized from ethyl acetate.

### Refinement

H atoms were positioned geometrically and refined as riding with C—H = 0.95 Å, and *U*_iso_(H) = 1.2*U*_eq_(C).

### Crystal data

**Table:**

C_14_H_4_Cl_4_FNO_2_	*F*(000) = 752
*M**_r_* = 378.98	*D*_x_ = 1.798 Mg m^−^^3^
Monoclinic, *P*2_1_/*c*	Mo *K*α radiation, λ = 0.71073 Å
Hall symbol: -P 2ybc	Cell parameters from 4679 reflections
*a* = 12.032 (2) Å	θ = 2.3–27.9°
*b* = 13.393 (3) Å	µ = 0.86 mm^−^^1^
*c* = 8.7244 (17) Å	*T* = 113 K
β = 95.33 (3)°	Prism, colorless
*V* = 1399.8 (5) Å^3^	0.22 × 0.20 × 0.16 mm
*Z* = 4	

### Data collection

**Table:**

Rigaku Saturn CCD area-detector diffractometer	2462 independent reflections
Radiation source: rotating anode	2120 reflections with *I* > 2σ(*I*)
confocal	*R*_int_ = 0.027
Detector resolution: 7.31 pixels mm^-1^	θ_max_ = 25.0°, θ_min_ = 2.3°
ω and φ scans	*h* = −14→13
Absorption correction: multi-scan (*CrystalClear*; Rigaku, 2005)	*k* = −15→15
*T*_min_ = 0.833, *T*_max_ = 0.875	*l* = −7→10
9870 measured reflections	

### Refinement

**Table:**

Refinement on *F*^2^	Primary atom site location: structure-invariant direct methods
Least-squares matrix: full	Secondary atom site location: difference Fourier map
*R*[*F*^2^ > 2σ(*F*^2^)] = 0.032	Hydrogen site location: inferred from neighbouring sites
*wR*(*F*^2^) = 0.085	H-atom parameters constrained
*S* = 1.14	*w* = 1/[σ^2^(*F*_o_^2^) + (0.0567*P*)^2^] where *P* = (*F*_o_^2^ + 2*F*_c_^2^)/3
2462 reflections	(Δ/σ)_max_ = 0.001
199 parameters	Δρ_max_ = 0.55 e Å^−^^3^
0 restraints	Δρ_min_ = −0.54 e Å^−^^3^

### Special details

**Table:**

Geometry. All e.s.d.'s (except the e.s.d. in the dihedral angle between two l.s. planes) are estimated using the full covariance matrix. The cell e.s.d.'s are taken into account individually in the estimation of e.s.d.'s in distances, angles and torsion angles; correlations between e.s.d.'s in cell parameters are only used when they are defined by crystal symmetry. An approximate (isotropic) treatment of cell e.s.d.'s is used for estimating e.s.d.'s involving l.s. planes.
Refinement. Refinement of *F*^2^ against ALL reflections. The weighted *R*-factor *wR* and goodness of fit *S* are based on *F*^2^, conventional *R*-factors *R* are based on *F*, with *F* set to zero for negative *F*^2^. The threshold expression of *F*^2^ > σ(*F*^2^) is used only for calculating *R*-factors(gt) *etc*. and is not relevant to the choice of reflections for refinement. *R*-factors based on *F*^2^ are statistically about twice as large as those based on *F*, and *R*- factors based on ALL data will be even larger.

### Fractional atomic coordinates and isotropic or equivalent isotropic displacement parameters (Å^2^)

**Table:**

	*x*	*y*	*z*	*U*_iso_*/*U*_eq_	
Cl1	0.86460 (4)	0.88811 (4)	0.87885 (5)	0.02115 (15)	
Cl2	1.12105 (4)	0.89461 (4)	0.84021 (6)	0.02482 (16)	
Cl3	1.20515 (4)	0.87608 (4)	0.51578 (6)	0.02211 (16)	
Cl4	1.03483 (4)	0.86419 (3)	0.22341 (5)	0.01784 (15)	
F1	0.59427 (10)	1.01280 (10)	0.21749 (17)	0.0449 (4)	
O1	0.64955 (11)	0.87452 (10)	0.64858 (16)	0.0242 (4)	
O2	0.77309 (11)	0.84862 (10)	0.17056 (15)	0.0222 (3)	
N1	0.68479 (12)	0.85955 (12)	0.39299 (18)	0.0179 (4)	
C1	0.71360 (15)	0.86955 (14)	0.5529 (2)	0.0175 (4)	
C2	0.83872 (15)	0.87331 (13)	0.5693 (2)	0.0145 (4)	
C3	0.91144 (15)	0.88264 (13)	0.6996 (2)	0.0159 (4)	
C4	1.02658 (15)	0.88421 (13)	0.6811 (2)	0.0157 (4)	
C5	1.06451 (15)	0.87662 (12)	0.5352 (2)	0.0158 (4)	
C6	0.98857 (15)	0.86904 (13)	0.4041 (2)	0.0140 (4)	
C7	0.87595 (15)	0.86712 (13)	0.4244 (2)	0.0143 (4)	
C8	0.77660 (15)	0.85734 (14)	0.3073 (2)	0.0162 (4)	
C9	0.57195 (15)	0.85497 (15)	0.3257 (2)	0.0196 (4)	
C10	0.50639 (15)	0.77220 (16)	0.3485 (2)	0.0252 (5)	
H10	0.5353	0.7180	0.4101	0.030*	
C11	0.39733 (16)	0.76994 (17)	0.2793 (2)	0.0315 (5)	
H11	0.3512	0.7139	0.2947	0.038*	
C12	0.35573 (17)	0.84825 (18)	0.1888 (3)	0.0318 (5)	
H12	0.2816	0.8452	0.1410	0.038*	
C13	0.42123 (16)	0.93161 (18)	0.1668 (2)	0.0313 (5)	
H13	0.3926	0.9862	0.1057	0.038*	
C14	0.52798 (15)	0.93280 (16)	0.2357 (2)	0.0243 (5)	

### Atomic displacement parameters (Å^2^)

**Table:**

	*U*^11^	*U*^22^	*U*^33^	*U*^12^	*U*^13^	*U*^23^
Cl1	0.0284 (3)	0.0246 (3)	0.0108 (3)	−0.00137 (19)	0.0036 (2)	−0.00012 (18)
Cl2	0.0242 (3)	0.0309 (3)	0.0178 (3)	0.0013 (2)	−0.0064 (2)	−0.0022 (2)
Cl3	0.0141 (3)	0.0256 (3)	0.0263 (3)	0.00246 (18)	0.0001 (2)	−0.00010 (19)
Cl4	0.0185 (3)	0.0213 (3)	0.0145 (2)	−0.00069 (17)	0.00566 (19)	−0.00100 (18)
F1	0.0410 (7)	0.0357 (8)	0.0559 (9)	−0.0095 (6)	−0.0078 (6)	0.0140 (7)
O1	0.0213 (7)	0.0350 (9)	0.0178 (7)	−0.0025 (6)	0.0088 (6)	−0.0029 (6)
O2	0.0208 (7)	0.0331 (8)	0.0127 (7)	−0.0050 (6)	0.0025 (5)	−0.0015 (6)
N1	0.0134 (8)	0.0252 (10)	0.0156 (8)	−0.0030 (6)	0.0037 (7)	−0.0021 (7)
C1	0.0196 (10)	0.0162 (11)	0.0166 (10)	−0.0009 (7)	0.0017 (8)	0.0000 (7)
C2	0.0181 (9)	0.0104 (10)	0.0155 (10)	−0.0004 (7)	0.0035 (8)	0.0016 (7)
C3	0.0235 (10)	0.0125 (10)	0.0119 (10)	0.0000 (7)	0.0032 (8)	−0.0001 (7)
C4	0.0198 (10)	0.0105 (10)	0.0157 (10)	−0.0005 (7)	−0.0044 (8)	0.0001 (7)
C5	0.0156 (9)	0.0106 (10)	0.0211 (11)	0.0014 (7)	0.0007 (8)	−0.0001 (7)
C6	0.0187 (9)	0.0107 (10)	0.0132 (9)	0.0001 (7)	0.0044 (8)	0.0012 (7)
C7	0.0174 (9)	0.0122 (10)	0.0129 (9)	−0.0015 (7)	0.0002 (7)	0.0000 (7)
C8	0.0166 (9)	0.0144 (10)	0.0176 (10)	−0.0026 (7)	0.0009 (8)	−0.0002 (8)
C9	0.0142 (9)	0.0269 (12)	0.0180 (10)	−0.0014 (8)	0.0030 (8)	−0.0057 (8)
C10	0.0208 (10)	0.0286 (13)	0.0270 (11)	−0.0041 (8)	0.0056 (8)	−0.0016 (9)
C11	0.0187 (10)	0.0390 (14)	0.0375 (13)	−0.0123 (9)	0.0064 (9)	−0.0100 (10)
C12	0.0149 (10)	0.0509 (16)	0.0295 (13)	−0.0006 (10)	0.0008 (9)	−0.0137 (11)
C13	0.0244 (11)	0.0406 (14)	0.0282 (12)	0.0073 (10)	−0.0012 (9)	−0.0020 (10)
C14	0.0231 (10)	0.0253 (12)	0.0243 (11)	−0.0040 (8)	0.0011 (8)	−0.0012 (9)

### Geometric parameters (Å, °)

**Table:**

Cl1—C3	1.7122 (19)	C4—C5	1.396 (3)
Cl2—C4	1.7159 (19)	C5—C6	1.400 (3)
Cl3—C5	1.7162 (18)	C6—C7	1.383 (3)
Cl4—C6	1.7213 (19)	C7—C8	1.505 (3)
F1—C14	1.354 (2)	C9—C14	1.380 (3)
O1—C1	1.189 (2)	C9—C10	1.385 (3)
O2—C8	1.195 (2)	C10—C11	1.393 (3)
N1—C8	1.391 (2)	C10—H10	0.9500
N1—C1	1.412 (2)	C11—C12	1.379 (3)
N1—C9	1.430 (2)	C11—H11	0.9500
C1—C2	1.500 (3)	C12—C13	1.390 (3)
C2—C3	1.374 (3)	C12—H12	0.9500
C2—C7	1.382 (3)	C13—C14	1.367 (3)
C3—C4	1.410 (3)	C13—H13	0.9500
			
C8—N1—C1	113.51 (15)	C6—C7—C8	129.85 (18)
C8—N1—C9	123.34 (16)	O2—C8—N1	125.63 (17)
C1—N1—C9	123.13 (16)	O2—C8—C7	129.69 (17)
O1—C1—N1	125.66 (17)	N1—C8—C7	104.68 (16)
O1—C1—C2	129.89 (18)	C14—C9—C10	119.51 (18)
N1—C1—C2	104.45 (16)	C14—C9—N1	120.02 (17)
C3—C2—C7	121.77 (18)	C10—C9—N1	120.46 (18)
C3—C2—C1	129.66 (18)	C9—C10—C11	118.8 (2)
C7—C2—C1	108.56 (16)	C9—C10—H10	120.6
C2—C3—C4	117.62 (18)	C11—C10—H10	120.6
C2—C3—Cl1	121.41 (15)	C12—C11—C10	120.6 (2)
C4—C3—Cl1	120.94 (15)	C12—C11—H11	119.7
C5—C4—C3	120.75 (17)	C10—C11—H11	119.7
C5—C4—Cl2	119.71 (14)	C11—C12—C13	120.64 (19)
C3—C4—Cl2	119.54 (15)	C11—C12—H12	119.7
C4—C5—C6	120.45 (17)	C13—C12—H12	119.7
C4—C5—Cl3	119.95 (15)	C14—C13—C12	118.1 (2)
C6—C5—Cl3	119.60 (15)	C14—C13—H13	121.0
C7—C6—C5	118.03 (18)	C12—C13—H13	121.0
C7—C6—Cl4	121.36 (15)	F1—C14—C13	119.8 (2)
C5—C6—Cl4	120.61 (14)	F1—C14—C9	117.75 (16)
C2—C7—C6	121.36 (17)	C13—C14—C9	122.4 (2)
C2—C7—C8	108.79 (16)		
			
C8—N1—C1—O1	178.91 (18)	C5—C6—C7—C2	0.5 (3)
C9—N1—C1—O1	0.6 (3)	Cl4—C6—C7—C2	−178.51 (13)
C8—N1—C1—C2	−0.4 (2)	C5—C6—C7—C8	−178.53 (17)
C9—N1—C1—C2	−178.80 (16)	Cl4—C6—C7—C8	2.4 (3)
O1—C1—C2—C3	0.6 (3)	C1—N1—C8—O2	179.37 (17)
N1—C1—C2—C3	179.94 (17)	C9—N1—C8—O2	−2.3 (3)
O1—C1—C2—C7	−178.47 (19)	C1—N1—C8—C7	−0.1 (2)
N1—C1—C2—C7	0.85 (19)	C9—N1—C8—C7	178.26 (16)
C7—C2—C3—C4	−0.9 (3)	C2—C7—C8—O2	−178.79 (19)
C1—C2—C3—C4	−179.94 (17)	C6—C7—C8—O2	0.4 (3)
C7—C2—C3—Cl1	−178.88 (14)	C2—C7—C8—N1	0.6 (2)
C1—C2—C3—Cl1	2.1 (3)	C6—C7—C8—N1	179.79 (18)
C2—C3—C4—C5	0.1 (3)	C8—N1—C9—C14	−67.0 (3)
Cl1—C3—C4—C5	178.00 (13)	C1—N1—C9—C14	111.2 (2)
C2—C3—C4—Cl2	−179.43 (13)	C8—N1—C9—C10	112.3 (2)
Cl1—C3—C4—Cl2	−1.5 (2)	C1—N1—C9—C10	−69.5 (2)
C3—C4—C5—C6	1.1 (3)	C14—C9—C10—C11	0.1 (3)
Cl2—C4—C5—C6	−179.40 (13)	N1—C9—C10—C11	−179.17 (18)
C3—C4—C5—Cl3	−178.64 (13)	C9—C10—C11—C12	0.6 (3)
Cl2—C4—C5—Cl3	0.9 (2)	C10—C11—C12—C13	−1.0 (3)
C4—C5—C6—C7	−1.4 (3)	C11—C12—C13—C14	0.9 (3)
Cl3—C5—C6—C7	178.36 (13)	C12—C13—C14—F1	−179.79 (18)
C4—C5—C6—Cl4	177.65 (13)	C12—C13—C14—C9	−0.2 (3)
Cl3—C5—C6—Cl4	−2.6 (2)	C10—C9—C14—F1	179.34 (18)
C3—C2—C7—C6	0.7 (3)	N1—C9—C14—F1	−1.4 (3)
C1—C2—C7—C6	179.84 (16)	C10—C9—C14—C13	−0.2 (3)
C3—C2—C7—C8	179.90 (16)	N1—C9—C14—C13	179.00 (19)
C1—C2—C7—C8	−0.92 (19)		
